# Investigating confounders of the association between survival and adjuvant radiation therapy after breast conserving surgery in a sample of elderly breast Cancer patients in Appalachia

**DOI:** 10.1186/s12885-019-6263-3

**Published:** 2019-12-17

**Authors:** Fabian Camacho, Roger Anderson, Gretchen Kimmick

**Affiliations:** 1 Department of Public Health Sciences, University of Virginia, Charlottesville, VA 22903 USA; 2 Duke Cancer Institute, Duke University, Durham, USA

## Abstract

**Background:**

To explain the association between adjuvant radiation therapy after breast conserving surgery (BCS RT) and overall survival (OS) by quantifying bias due to confounding in a sample of elderly breast cancer beneficiaries in a multi-state region of Appalachia.

**Methods:**

We used Medicare claims linked registry data for fee-for-service beneficiaries with AJCC stage I-III, treated with BCS, and diagnosed from 2006 to 2008 in Appalachian counties of Kentucky, Ohio, North Carolina, and Pennsylvania. Confounders of BCS RT included age, rurality, regional SES, access to radiation facilities, marital status, Charlson comorbidity, Medicaid dual status, institutionalization, tumor characteristics, and surgical facility characteristics. Adjusted percent change in expected survival by BCS RT was examined using Accelerated Failure Time (AFT) models. Confounding bias was assessed by comparing effects between adjusted and partially adjusted associations using a fully specified structural model.

**Results:**

The final sample had 2675 beneficiaries with mean age of 75, with 81% 5-year survival from diagnosis. Unadjusted percentage increase in expected survival was 2.75 times greater in the RT group vs. non-RT group, with 5-year survival of 85% vs 60%; fully adjusted percentage increase was 1.70 times greater, with 5-year rates of 83% vs 71%. Quantification of incremental confounding showed age accounted for 71% of the effect reduction, followed by tumor features (12%), comorbidity (10%), dual status(10%), and institutionalization (8%). Adjusting for age and tumor features only resulted in only 4% bias from fully adjusted percent change (70% change vs 66%).

**Conclusion:**

Quantification of confounding aids in determining covariates to adjust for and in interpreting raw associations. Substantial confounding was present (60% of total association), with age accounting for the largest share (71%); adjusting for age plus tumor features corrected for most of the confounding (4% bias). The direct effect of BCS RT on OS accounted for 40% of the total association.

## Background

Radiation therapy (RT) after breast conserving surgery (BCS) in non-metastatic breast cancer (BC) patients has been shown in clinical studies to improve long term (> 10 years) survival [1–4], compared to BCS alone. Thus adjuvant RT has been a consistent recommendation in standard of care guidelines for Stage I-III patients undergoing BCS [5–7]. Analysis of registry data, at the population level, suggests even larger survival benefits of RT [8, 9] and detectable differences in short term survival (< 6 years), which remains in certain subpopulations even after adjusting for relevant confounders [10–12].

A major challenge of the latter real-word or “effectiveness “studies is to explain the magnitude of the association of a recommended therapy, such as RT, on survival apart from the influence of important confounding factors, which often include comorbidity, access to care, socio-economic status, and quality of care [10, 11]. Such a quantification of confounding may be important in determining whether a covariate needs to be adjusted for in an analysis and aid in the interpretation of unadjusted associations [13].

In this study, we document the effect of adjuvant RT on survival to model and quantify the magnitude of the benefit from treatment that may be due to significant confounding. Methods for quantifying confounding have been well-documented which enable investigation of confounding bias [13] and which are implemented in this paper. The study population is a sample of a mostly elderly population residing in Appalachian counties of 4 states (Pennsylvania, Ohio, Kentucky, North Carolina) as defined by the Appalachian Regional Commission and chosen to capture the breadth of the Appalachian region. This geographical region has higher cancer incidence and mortality rates [14–16], applicable to breast cancer mortality as well [17], with heterogeneous economic diversity [18], poor health care accessibility [18, 19] significant medically underserved pockets [18], and is distinct from regions from studies using SEER (Surveillance, Epidemiology, and End Results Program)-Medicare linked databases [20–22].

## Methods

### Study population

Female sample beneficiaries with breast cancer and diagnosed from 2006 to 2008 resided in the four state region of Appalachia defined by the Appalachian Regional Commission (see Additional file 1 map, section 3). Beneficiary characteristics were extracted from each state cancer registry. Beneficiaries were then linked to their corresponding Medicare claims from 2005 to 2009 by matching social security number, birth date, and gender. Conforming to published inclusion criteria [23], beneficiaries were restricted to having diagnostically confirmed breast cancers, fee for service (FFS) continuous Medicare enrollment 1 year after and before diagnosis, a first cancer diagnosis, no multiple/concurrent cancers within 90 days, 1 year survival after cancer diagnosis, AJCC (American Joint Committee on Cancer) stage I-III, and BCS during 6 months after diagnosis.

### Study variables

Overall survival was derived from a composite of updated registry NDI (National Death Index) survival and Medicare beneficiary death date updated until Dec 2014. Identification of BCS was based on registry primary site surgery variable supplemented by Medicare claims using a study-specific algorithm [23]. Codes used included registry site-specific codes (10–20), ICD-9-CM procedure codes 85.20–8.23, 86.25, and HCPCS/CPT codes 19,120, 19,125, 19,126, 19,160, 19,162, 19,301, 19,302 [23]. Identification of Adjuvant RT was based on a registry radiation sequence variable supplemented by Medicare claims using look-up tables provided by the NCI (National Cancer Institute) [24]. RT was assumed given if radiation codes appeared for 15 days after surgery during the year after diagnosis.

Geographical variables included county level measures for rurality based on the US Department of Agriculture 2013 Beale codes [25] and an area deprivation index (Singh) based on 2000 census measures [26]. The number of hospitals/health care systems with radiation services within a 50 mile straight-line radius from patient residence was calculated as a measure of accessibility, as determined from AHA (American Hospital Association) annual 2010 survey database [27]. The 50 mile cut-off was chosen based on choosing the cut-off with strongest prediction of RT delivery, based on fitting a logistic regression with RT delivery rate as a function of successively increasing distance thresholds ranging from 25 to 100 miles in 5 mile increments.

Potential confounders included beneficiary comorbidity, enrollment in Medicaid during year after diagnosis, and being institutionalized. Comorbidity was based on a Charlson-Deyo score [28, 29] calculated during the year prior to diagnosis. Institutionalization was assessed if either at least two claims existed with shared living, nursing/custodial facility, or hospice, or beneficiary had more than 15 days in a skilled nursing facility during the year after diagnosis. Other confounders consisted of age at diagnosis, marital status (single vs married), AJCC Derived Stage (I,II,III), first cancer, grade, histology, positive lymph nodes, ER/PR (Estrogen/Progesterone) positive tumor, tumor size, total number of beds and therapeutic radiology services of surgical facility [23], facility accreditation with the COC (Commission of Cancer) obtained from a web site locator during 2011 [30], and surgical provider volume calculated from claims during the calendar year 2008.

Although RT after BCS has been viewed as guideline for all cases [6, 7], other authors suggest RT as optional for subgroups of elderly patients, such as patients jointly aged ≥70 years, with ER/PR positive, < 2 cm, and node negative tumors [23]. These recommendations are based on findings documenting lower benefit of RT in these subgroups [31, 32] . As a result, an indicator for optional RT was included as a potential confounder.

### Statistical methods

The primary effect of interest is percent change in expected survival time (E[T]) between survival for patients if they receive BCS-RT vs if they do not receive BCS-RT (δ = (E[T(1)] - E[T(0)]) / E[T(0)]). Confounding bias is defined as the difference (∆) between the marginal association δ_m_ (E[T|X = 1] - E[T|X = 0]) /E[T|X = 0] and δ [13]. Incremental confounding (IC) is the decomposition of this bias into contributions from each confounder.

Several methods have been suggested to estimate δ, ∆, and IC, which include standardization and Inverse Probability of Treatment Weights (IPTW) [13]. However, a regression based approach, is an alternative. Advantages of this method include its similarity to traditional regression techniques. Also, δ, ∆, and IC can be calculated under the presence of interactions with the main exposure. Doubly robust approaches combining IPTW and regression simultaneously may further protect against misspecification [33]. However, the statistical properties in the context of censored survival data and confounding quantification remains to be studied and is not covered in this paper.

**Fig. 1:**
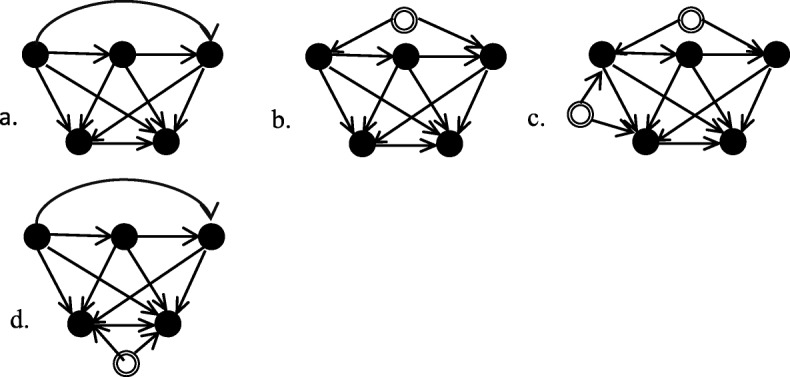
Directed Acyclic Graphs for Confounding Quantification. Shows causal graphs under which quantification is applicable or not applicable

Directed Acyclic Graphs (DAGS) (Fig. 1) are first presented to clarify the causal structure between variables. Figure 1 a describes the assumed causal network, where the topmost layer of nodes represents confounder variables ordered from antecedent to subsequent in a causal chain, and the bottom layer describes nodes representing the exposure (BCS-RT) and outcome (Survival). The quantification method successively conditions on confounders and uses the conditional associations between exposure-outcome. Graphically, pathways leading through conditioned nodes cease to contribute to the association, allowing for progressive elimination of pathways until a direct causal effect can be estimated [34]. The method of successive conditioning is applicable even if latent variables affect or are affected by the confounders, as illustrated in Fig. 1 b. One exception is shown in Fig. 1 c, where latent variables simultaneously affect one of the confounders, leading to an additional non-causal bias. Similarly, omitting important confounders (Fig. 1 d), results in biased effects.

Once the causal network has been considered, a parametric Accelerated Failure Time (AFT) model linking predictors to overall survival time was fit to the data. Although AFT models are a less applied alternative to the more popular Cox Proportional Hazards model [35, 36], an advantage is that log survival times are regressed directly on a linear combination of predictors, with an error term commonly following a log logistic, Weibull, or an exponential distribution. Percentage change in survival time compared to reference value can be calculated from the regression weights [37, 38]. Effects were considered statistically significant if False Discovery Rate (FDR) adjusted p-values were < .05 [39].

We sought to reduce several sources of misspecification bias for the full model. Estimation bias due to misspecification of the error distribution was addressed by choosing the model with lowest AIC (Akaike Information Criterion) [40]. A second source of bias exist if effect heterogeneity was present. Initially, a main effects AFT model was fit assuming the effect represents an average across covariate levels. Effect heterogeneity was then investigated through subgroup analysis, examining the effect of RT across all covariate levels; interaction terms were added to the main effects model if their separate interaction test *p*-values < .05. Other sources of bias concerned the possibility of misclassification error, which is not considered in this paper, and endogeneity bias, where the error term is correlated with the exposure. For the fully adjusted model, we expect the error term to be uncorrelated under the assumption that no residual confounding is present. Finally, non-informative censoring was assumed.

The next step consisted in estimating the effects δ, δ_m_, and partially adjusted effects δ_Z_ used to assess incremental confounding. These quantities were estimated using estimates from the fully adjusted and partially adjusted models. The partially adjusted models retained the same specification as the fully adjusted model but without variables that were not adjusted for. More detail is provided in the Additional file 1, section 1. From these quantities, overall ∆ and decomposition of ∆ into increments were calculated, where each increment assessed the change in confounding bias. Confidence intervals were calculated by non-parametric bootstrapping (1000 resamples) with replacement and using percentile method to determine endpoints.

Model based survival curves after confounder adjustment were calculated using the “direct adjusted” method [41–43] which averaged predicted survival from the fully specified model at each time point for each patient. In addition, Kaplan Meier (KM) and unadjusted model-based curves based on an unadjusted AFT model assessing the uncontrolled association between BCS-RT survival were calculated.

In order to treat missing data, multiple imputation (MI) was conducted using the method by White and Royston [44], which adds the cumulative hazard and censoring variable to the imputation model. Missing values were then imputed using the FCS (Fully Conditional Specification) method available in the SAS procedure MI, v9.3. To improve compatibility of the imputation model with the analysis model with interactions, the MI procedure was implemented separately on RT strata. In order to integrate MI into the calculation of confidence intervals, the data was bootstrapped first and MI was conducted for each bootstrap, as recommended in [45].

## Results

The final sample consisted of 2675 beneficiaries. From Table 1, the average age was 75, most patients lived in metropolitan regions (59%), average number of local radiation facilities was 5, average Charlson score was 1.34 (standard deviation = 1.62), only 15% where Medicaid duals, 6% were institutionalized, 70% were Stage I, 85% had first cancer tumors, 50% had moderately differentiated grade, 92% had Ductal/tubular histology, 85% were lymph node negative, 88% were ER/PR positive, average tumor size was 1.6 cm, 71% patients had surgical treatment in a COC designated facility, with average number of 339 beds, and 77% of such facilities offered radiation services. Lastly, 56% did not meet optional radiation therapy criteria.

**Table 1:** Characteristics and Predictors of Adjuvant Radiation Therapy (*N* = 2675)

Overall	N (%)		N(%)
Age Quartile		Grade^§^	N_miss_ = 158
Q1 [≤69]	700 (26.2)	Well differentiated	712 (28.3)
Q2 [70–75]	701 (26.2)	Moderately differentiated	1203 (47.8)
Q3 [76–80]	609 (22.8)	Poor/Undifferentiated	602 (23.9)
Q4 [81+]	665 (24.9)	Histology	
Mean (Std) [IQR]	74.8 (8.4) [11]	Tube/colloid	138 (5.2)
Rural Status		Other	74 (2.8)
Metro	1584 (59.2)	Ductal/lobular	2463 (92.1)
Urban	967 (36.2)	Lymph Nodes	
Rural	124 (4.6)	Positive	413 (15.4)
Singh Index		Negative	2262 (84.6)
Q1 [Highest SES]	687 (25.7)	ER/PR positive	N_miss_ = 99
Q2	674 (25.2)	Positive	2264 (87.9)
Q3	649 (24.3)	Negative	312 (12.1)
Q4 [Lowest SES]	665 (24.9)	Tumor Size	N_miss_ = 88
Mean (Std) [IQR]	86.3 (13.3) [16]	Q1 [≤ 9 mm]	708 (27.4)
Access to Near Radiation Facilities		Q2 [10–13]	601 (23.2)
Q1 [≤2]	1056 (39.5)	Q3 [14–20]	727 (28.1)
Q2 [3–4]	497 (18.6)	Q4 [20+]	551 (21.3)
Q3 [5]	530 (19.8)	Mean(Std)[IQR]	15.7 (11.5) [11]
Q4 [6+]	592 (22.1)	COC status	
Mean (Std) [IQR]	4.7 (4.7) [3]	Yes	1898 (71.0)
Region		No	777 (29.1)
Kentucky	251 (9.4)	SF Number of Beds	N_miss_ = 32
North Carolina	504 (18.8)	Mean (Std) [IQR]	339 (244) [279]
Ohio	525 (19.6)	SF Radiation Services	N_miss_ = 45
Pennsyvlania	1395 (52.2)	Yes	2031 (77.2)
Marital Status	N_miss_ = 72	No	599 (22.8)
Single	1390 (53.4)	Optional RT	N_miss_ = 176
Married	1213 (46.6)	Yes	1098 (43.9)
Charlson Comorbidity Score		No	1401 (56.1)
0	1014 (37.9)	Surg Provider Volume	N_miss_ = 134
1	805 (30.1)	Mean (Std) [IQR]	16.2 (20.3) [20]
2+	856 (32.0)	Adjuvant Radiation Therapy	
Meand (Std) [IQR]	1.34 (1.62) [2]	Yes	2128 (79.6)
Medicaid Dual		No	547 (20.5)
Yes	387 (14.5)	OS Survival in months^§§^	
No	2288 (85.5)	Observed Mean	5.5 (1.9) [2.0]
Institutionalized			
Yes	163 (6.1)		
No	2512 (94.9))		
Stage			
Stage I	1869 (69.9)		
Stage II	697 (26.1)		
Stage III	109 (4.1)		
First Cancer			
Yes	2285 (85.4)		
No	390 (14.6)		

**Fig. 2:**
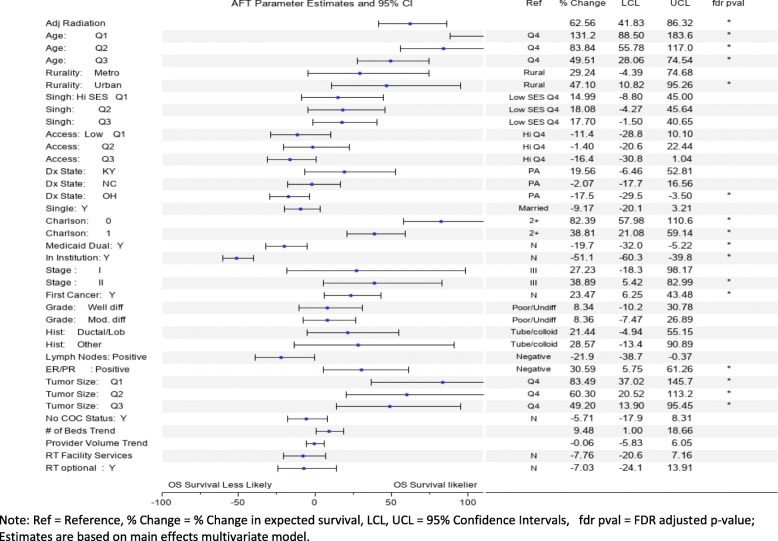
Predictors of OS survival. Shows parameter estimates for AFT multivariate model where adjuvant radiation and other covariates predict overall survival

**Fig. 3:**
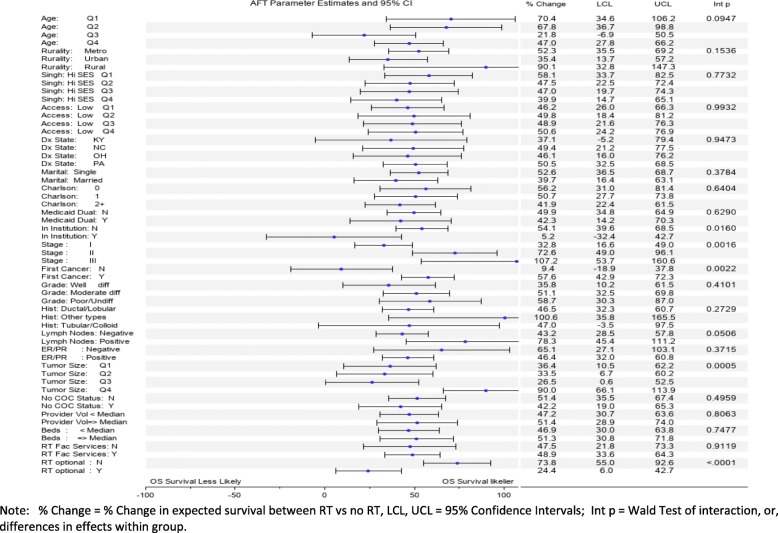
Adjuvant Radiation Estimates by subgroup analysis. Shows parameter estimates for subgroup analysis

Significant predictors of increased OS based on the fully adjusted main effect AFT model, using percent change (% CH) in expected survival as a measure of effect (Fig. 2), included receiving RT (% CH = 63), younger age (Q1,Q2,Q3 vs Q4, %CH = 131,84,50), urban residence (vs rural, %CH = 47), lower Charlson comorbidity (0,1 vs 2+ vs 3, %CH = 82, 39), stage II (vs III, % CH = 39), no prior cancer diagnosis (%CH = 23), ER/PR positive (vs negative, %CH = 31), smaller tumor size (Q1, Q2, Q3 vs Q4, %CH = 83,60,49). Decreased OS was predicted by OH residence (vs PA, % CH = − 18), Medicaid Dual insurance status (% CH = − 20), and being institutionalized (% CH = − 51). Figure 3 shows results from the subgroup analysis. Variations, or heterogeneity, in the RT effect size, was detected for being institutionalized (% CH Yes = 5 vs No = 54, *p* = .0160), stage (% CH I = 33, II = 73, III = 107, *p* = .0016), first cancer (% CH Yes = 58 vs No = 9, *p* = .0022), lymph nodes (% CH Positive = 78 vs Negative = 43, *p* = .0506), tumor size (% CH Q1 = 36, Q2 = 33, Q3 = 27, Q4 = 90, *p* = .0005), and optional RT (% CH optional Y = 24, *N* = 74, *p* < .0001).

**Fig. 4:**
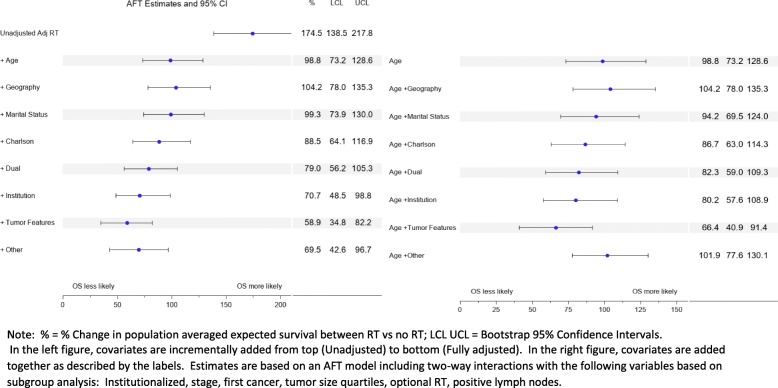
Partially and fully adjusted RT population averaged % change. Shows confounder quantification metrics

Figure 4 shows the quantification of confounding based on comparing population averaged expected survival change (% CH), where survival is measured starting 1 year after diagnosis. The unadjusted % CH for RT = 175%, suggesting expected survival for those who receive RT is 2.75 times greater than those who were not treated with RT, whereas the fully adjusted % CH based on a model with interactions from subgroup analysis was % CH = 70%, or an expected survival 1.70 times greater with RT (*p* < .0001). The difference between these two quantities was statistically significant (∆ = 105, bootstrap 95 CI% 79,143).

When examining incremental confounding differences compared to total differences in annual survival using the default sequential order (left chart in Fig. 4), age accounts to 72% of the difference, there is a 5% increase due to adding geography, 5% reduction from marital status, 10% reduction from comorbidity, 10% reduction due to adding dual Medicaid/Medicare status, 8% reduction due to adding institutionalization, 12% reduction due to adding tumor features, and 11% increase by adding the remaining features. With the exception of institutionalization, all successive confounding differences were significant based on overlap of the 95% bootstrap confidence intervals with zero.

When confounders are separately added after adjusting for age (right chart in Fig. 4), all except for geography and ‘other’ contribute towards bias reduction. These include marital status (76% reduction) comorbidity (84%), dual (88%), institutionalization (90%), and tumor features (103%). In particular, adding age plus tumor features only results in an % change effect of 66.4% which, when compared to the fully adjusted effect of 69.5, is off by a 4% bias.

**Fig. 5:**
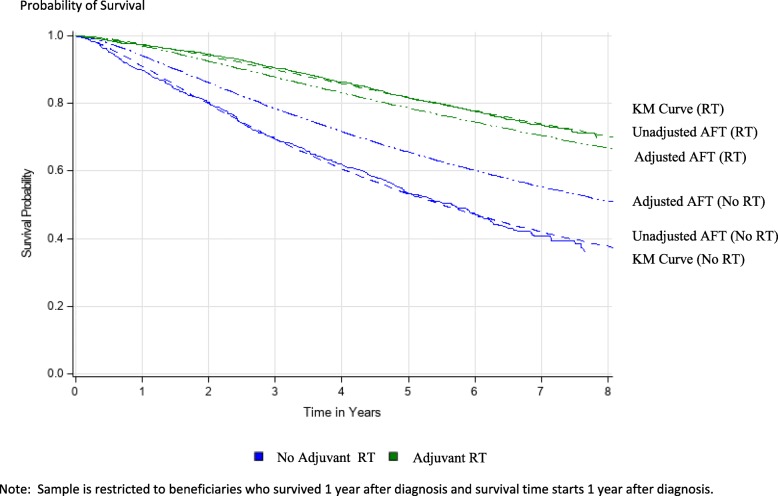
Overall Survival Curves. Shows adjusted and unadjusted survival curves

Figure 5 shows KM and AFT model-based unadjusted/adjusted survival curves stratified by RT and no RT cohorts. The AFT curves are presented by dashed lines and KM curves are continuous. As a diagnostic of goodness of fit, the model-based unadjusted curves closely track the KM curves. Unadjusted 5 year OS rates were 60% vs 85%, compared to adjusted OS rates of 71% vs 83%, for those receiving RT after BCS vs BCS alone.

## Discussion

In a typical population based patterns of breast cancer care study, we quantified significant confounding of the association between use of RT after BCS and survival. Confounding variables accounted for approximately half of the survival benefit of having received RT after BCS vs not. This large magnitude of confounding warrants caution in interpretation of treatment benefits in population-based studies. While the specific set of confounders that an investigator chooses to examine will depend upon various study attributes, and could differ from ours, we nonetheless sought to test a set of plausible confounders relevant to health services research and health disparities in breast cancer treatment in an elderly sample.

While adjusting for confounding factors in analyzing the effect of BCS RT on survival is standard practice, there are relatively few modern papers in oncology and health disparities that examine and demonstrate the magnitude of this source of bias. Prior studies have hypothesized on possible mechanisms which may account for unanticipated higher mortality rates in women who forego RT after BCS, ranging from comorbidity, poverty, and lack of access [11], with particular suspicion centering on comorbidity and disability [23]. Our analysis suggests that, while comorbidity and associated measures may contribute towards reducing confounding bias incrementally and individually, adjusting for age, and tumor characteristics only as performed in most epidemiologic and randomized studies [46–48] may account for most of the bias (66% change vs 70% change, 4% bias, Fig. 4).

Age significantly had the most contribution to confounding (71%). Although this finding may not be noteworthy given that age is a standard covariate in survival analysis, the unexpected magnitude of contribution suggests unadjusted RT survival curves may be subject to substantial bias if not controlled for age.

Appalachian regions varied considerably by socio-demographic characteristics. Most notably, patients in KY lived in significantly more rural areas than PA (20% vs 1%, *p* < .0001, using county-level USDA Beale codes) with the average number of nearby radiation facilities within a 50-mile straight-line radius of patient residence in KY being significantly lower than PA (1.31 vs 6.2, *p* < .0001), and with significantly lower regional SES (*p* < .0001) as well. Patients living in KY and NC were significantly less likely than PA to receive RT by approximately 6 percentage points. However, the addition of geographic variables to the age adjusted model resulted in an increase in the association of RT on OS. This result is explained by a pattern of better survival in KY compared to other states. For example, a percent change in survival of 20.6% vs PA, 46.0% vs OH, and 22.7% vs NC was observed under the main effects model. This was unexpected given KY’s rurality. After adjusting for state of residence, the other geographical measures, including health care access, SES (Socioeconomic Status), and rurality, are not related to receipt of RT and thus do not contribute to incremental confounding.

Consistent with comorbidity being hypothesized as an important confounder [10], its contribution to incremental confounding ranks was positive (11%) although not as strong as we expected. Confounding may occur because higher comorbidity coincides with increased risk of death and at the same time women who have more comorbidity are less likely to receive RT. Furthermore, this contribution may be stronger than in other samples given that the generally higher rates of comorbidity in rural Appalachia than more affluent areas of the United States may increase the importance of this source of confounding [49, 50]. Alternatively, it is possible the Charlson-comorbidity score does not fully capture a patient’s level of illness unrelated to cancer as similar samples have reported lower rates of no-comorbidity [51].

A related health status confounder was institutionalization assessed as ongoing care in a skilled nursing facility or receipt of domiciliary care. While few population-based patterns of care studies include measures of this status in their models, our findings suggest that it is a potential source of confounding with regard to access to cancer treatment and survival (8% in incremental confounding using default order, 18% when age is included only, and 90% jointly with age). Institutionalized breast cancer patients may suffer from increased barriers to treatment and should be considered in multivariate models comparing treatments or populations.

Medicaid dual status, as an indicator of means-tested poverty, also contributed to reduction of confounding bias. The study region includes extremes of highly concentrated poverty in isolated rural areas of Appalachia to more affluent regions in PA and urbanized areas. The effect of poverty on access to breast cancer care and on outcomes is well documented in the literature [52–54] and is commonly included, along with age, in analytical models. The fully adjusted model included both area poverty (Singh index) and individual-level poverty assessed by dual status; both predicted access to RT (not shown) but only the latter was a significant source of confounding in the relationship of RT to OS.

We considered including concomitant treatments, such as use of adjuvant endocrine therapy (AET) in the survival model, but made the decision not to for three main reasons. First, the analysis would have been limited by the availability of pharmacy information for only two thirds of the sample. Secondly, it was unclear in our estimation whether such treatments are confounders, along confounding pathways, or mediators of the association between RT and survival, in which case the fully adjusted effect would be an over-controlled effect. Lastly, in the case of AET, such therapy is restricted primarily to ER/PR positive tumors and was not found to be predictive of survival in this stratum after fully adjusting for the other variables (% change = 13, Not Significant), possibly due to small sample size, low-risk observational populations, and too short follow-up to observe survival benefit.

The quantification approach assumes the data generating mechanism is a linear main effect AFT model with potential two-way interactions between exposure (RT) and covariates, which is a common assumption made when modelling using regression analysis. In addition failure to satisfy the backdoor criterion when conditioning on confounders may bias the estimates and may occur as a result of measurement error in the exposure/covariates or presence of hidden latent confounders. Furthermore, incremental confounding contributions may change depending on the order of decomposition, though we think our order is desirable as it follows antecedent causality (i.e. age affects comorbidity and not vice-versa).

Informally, we view the strength of confounding as a product of the association between the confounder with receipt of RT and its association with survival. Although the generalizability of the decomposition in other samples may be affected due to variations in these associations, we hypothesize the relative ordering and magnitudes will not be substantial in population based samples, such as this one involving elderly beneficiaries in the United States. In such is the case, findings and implications will then be applicable to similar settings.

## Conclusions

We explored the contribution of confounding variables to the unexpected, short-term OS benefit of the addition of RT to BCS versus BCS alone seen in epidemiologic studies. Quantification of confounding aids in determining covariates to include in a multivariate model and in interpreting raw associations. Substantial confounding was present (60% of total association), with age accounting for the largest share (71%); adjusting for age plus tumor features corrected for most of the confounding, resulting in only a 4% bias. The direct effect of having received RT after BCS on OS, however, seemed to account for 40% of the benefit.

## Supplementary information

**Additional file 1: Quantification of Confounding Bias.** Provides details on the quantification method. **Inclusion Criteria.** Graphically shows the inclusion criteria. **Map of Study Region.** Shows the Appalachian counties where patients were selected from. The map was created for the Appalachia Patterns of Care grant and intended for use by the Principal Investigators, including the authors.

## Data Availability

The datasets generated and analyzed during the current study are not publicly available due privacy restrictions placed by the data supplier (CMS, Centers for Medicare and Medicaid Services) and agreements with local registries. Please contact the first author F. Camacho for further details and access to the data.

## Abbreviations

% CH: Percent change
AET: Adjuvant Endocrine Therapy
AFT: Accelerated Failure Time
AHA: American Hospital Association
AIC: Akaike Information Criterion
AJCC: American Joint Committee on Cancer
BCS: Breast conserving surgery
COC: Commission of Cancer
CPT: Current Procedural Terminology
DAGS: Directed Acyclic Graphs
E[T]: Expected survival time
ER/PR: Estrogen/Progesterone Receptor
FCS: Fully Conditional Specification
FFS: Fee for Service
HCPCS: Healthcare Common Procedure Coding System
IC: Incremental Confounding
ICD-9: International Classification of Diseases, Ninth Revision, Clinical Modification
IPTW: Inverse Probability of Treatment Weights
KM: Kaplan Meier
KY: Kentucky
MI: Multiple Imputation
NC: North Carolina
NCI: National Cancer Institute
NDI: National Death Index
NS: Not Significant
OH: Ohio
OS: Overall Survival
PA: Pennsylvania
RT: Radiation therapy
SAS: Statistical Analysis System
SEER: Surveillance, Epidemiology, and End Results Program
SES: Socioeconomic Status
USDA: US Department of Agriculture
