# Supplementary material for: Investigating confounders of the association between survival and adjuvant radiation therapy after breast conserving surgery in a sample of elderly breast Cancer patients in Appalachia
Source: BMC Cancer. 2019 Dec 17;19:1228. doi: 10.1186/s12885-019-6263-3 (PMC6918701; doi:10.1186/s12885-019-6263-3)
Supplement: Supplementary file 1 — Additional file 1: Quantification of Confounding Bias. Provides details on the quantification method. Inclusion Criteria. Graphically shows the inclusion criteria. Map of Study Region. Shows the Appalachian counties where patients were selected from. The map was created for the Appalachia Patterns of Care grant and intended for use by the Principal Investigators, including the authors. [file 12885_2019_6263_MOESM1_ESM.pdf]

Appendix

1 Quantification of Confounding Bias

Using the well-established potential outcomes framework popularized by Rubin, we seek to estimate a causal effect of treatment on survival time T by comparing expected survival times $E[T(0)]$ and $E[T(1)]$ over the population, where $T(x)$ represents a patient's survival time if treatment is received ($x = 1$) and not received ($x = 0$).

Firstly, a structural model for the survival distribution was determined by an AFT model ('fully adjusted') according to the following equation:

$$\ln T = \beta_0 + \beta_1(\boldsymbol{Z})X + \boldsymbol{\beta Z} + \sigma\epsilon \tag{1}$$

where $\boldsymbol{\beta Z}$ is a linear combination of covariates $Z_1, Z_2, ..., Z_n$ and β_1 is a linear combination of $\boldsymbol{Z}$, allowing for effect modification of $\boldsymbol{Z}$. Under the consistency assumption,

$$E[T|X = x, \boldsymbol{Z}] = E[T(x)|X = x, \boldsymbol{Z}]. \tag{2}$$

Under the conditional ignorability assumption that implies no residual confounding is present after accounting for $\boldsymbol{Z}$, $T(x)|\boldsymbol{Z}$ independent of $X|\boldsymbol{Z}$. It follows

$$E[E[T|X = x, \boldsymbol{Z}]] = E[E[T(x)|\boldsymbol{Z}]] = E[T(x)]. \tag{3}$$

In order to estimate $E[T(x)]$, each observation in the data set was assigned two scores for $E[T|X = x, Z_1, ..., Z_n]$, setting X to 0 and 1 respectively. These scores were calculated using parameter estimates from the structural model. Scoring procedures are available in several statistical packages. For example, in SAS, proc lifereg output statement can output quantile estimates such as the median survival time for each observation. For log-logistic distributions, the median is proportional to the expected value, where the constant of proportionality depends on σ. Then the following approximation was used:

$$E[T(x)] = E[E[T|X = x, Z_1, ..., Z_n]] \approx \frac{1}{N}\sum E[T|X = x, Z_1, ..., Z_n] \tag{4}$$

, taking the average across the entire sample. The population effect in terms of percent change,

$$\delta = \frac{E[T(1)] - E[T(0)]}{E[T(0)]} \tag{5}$$

,was then estimated using the plug-in estimators for $E[T(x)]$.

Confounding bias ($\mathit{\Delta}$) is defined as the difference between δ and the marginal association $\delta_M = (E[T|X = 1] - E[T|X = 0])/E[T|X = 0]$. Estimation of δ_M was conducted by using a plug-in estimator of $E[T|X = x]$ based on an unadjusted AFT model: $\ln T = \beta_0 + \beta_1 \mathrm{X} + \sigma\epsilon$.

The problem of incremental confounding, which allows for decomposition of the bias into contributions by each confounder, if discussed in [1] and covers

two methods. Using the first method, the marginal association removing influence of confounders is estimated, say Z_1 and Z_2 for simplicity ($\delta^*_{Z_1Z_2}$). This association has the same formula as δ, except the expected values are based on $E[E[T|X, Z_1, Z_2]]$.

In order estimate these expected values, all the observations in the sample were scored twice with $E[T|X, Z_1, Z_2]$, setting X to 0 and 1. These expected values were calculated by using partially adjusted models following the following equations: $\ln T = \beta_0 + \beta_1(\boldsymbol{Z_j})X + \boldsymbol{\beta} \boldsymbol{Z_j} + \sigma\epsilon$, where $\boldsymbol{Z}_j$ is a subset of $\boldsymbol{Z}$; specifically for the above example, $\boldsymbol{Z}_j = Z_1$,Z_2. Interactions between $\boldsymbol{Z}_j$ and β_1 were retained if included in the full model.

Sample averages were then used to approximate $E[E[T|X, Z_1, Z_2]]$ and $\delta^*_{Z_1Z_2}$. Incremental confounding was then assessed by the difference $\delta^*_{Z_1Z_2}$ - $\delta^*_{Z_1}$ and interpreted as the change to confounding bias by accounting jointly for Z_1, Z_2 compared to Z_1 alone. Overall bias Δ can be decomposed into a sum of incremental differences, where each difference is derived by successively augmenting the list of confounders (i.e $\delta^*_{Z_1Z_2Z_3}$ - $\delta^*_{Z_1Z_2}$).

Under the second method, the expected conditional association removing the influence of successive confounders is estimated, for example $E[\delta^*_{Z_1Z_2}|Z_1]$, where $\delta^*_{Z_1Z_2}|Z_1$ has the same formula as δ except the expected values are based on $E[E[T|X, Z_1, Z_2]|Z_1]$. Then $E[\delta^*_{Z_1Z_2}|Z_1]$ is compared to the expected conditional association $E[\delta^*_{Z_1}|Z_1]$ to assess the amount of confounding bias removed by Z_2 conditional on Z_1.

When the effect measure (δ) is a difference between potential outcomes of X =1 and 0 both methods yield equivalent incremental differences [1] . However, in general this is not the case, such as in this presentation, where the effect measure is a ratio. A decomposition of confounding bias using the second method is in terms of incremental differences is not easily derivable in our opinion. For this reason, the first method was applied instead.

2 Inclusion Criteria

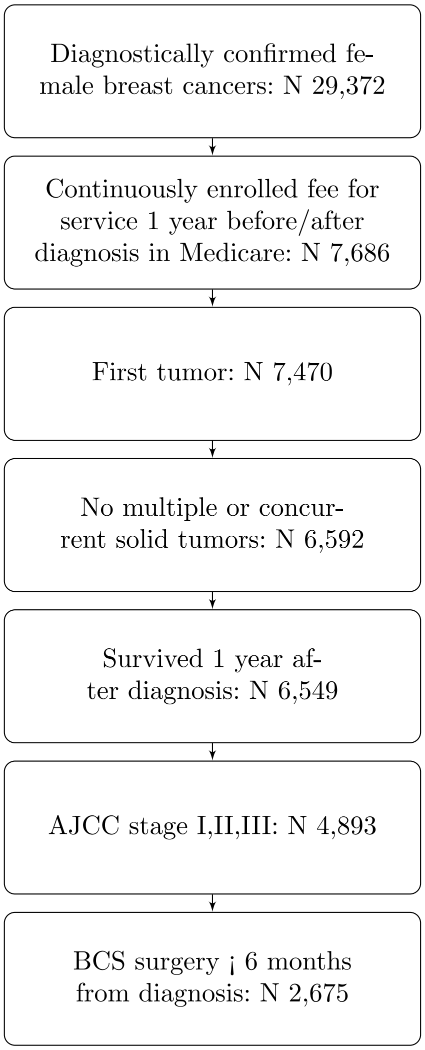

3 Map of Study Region

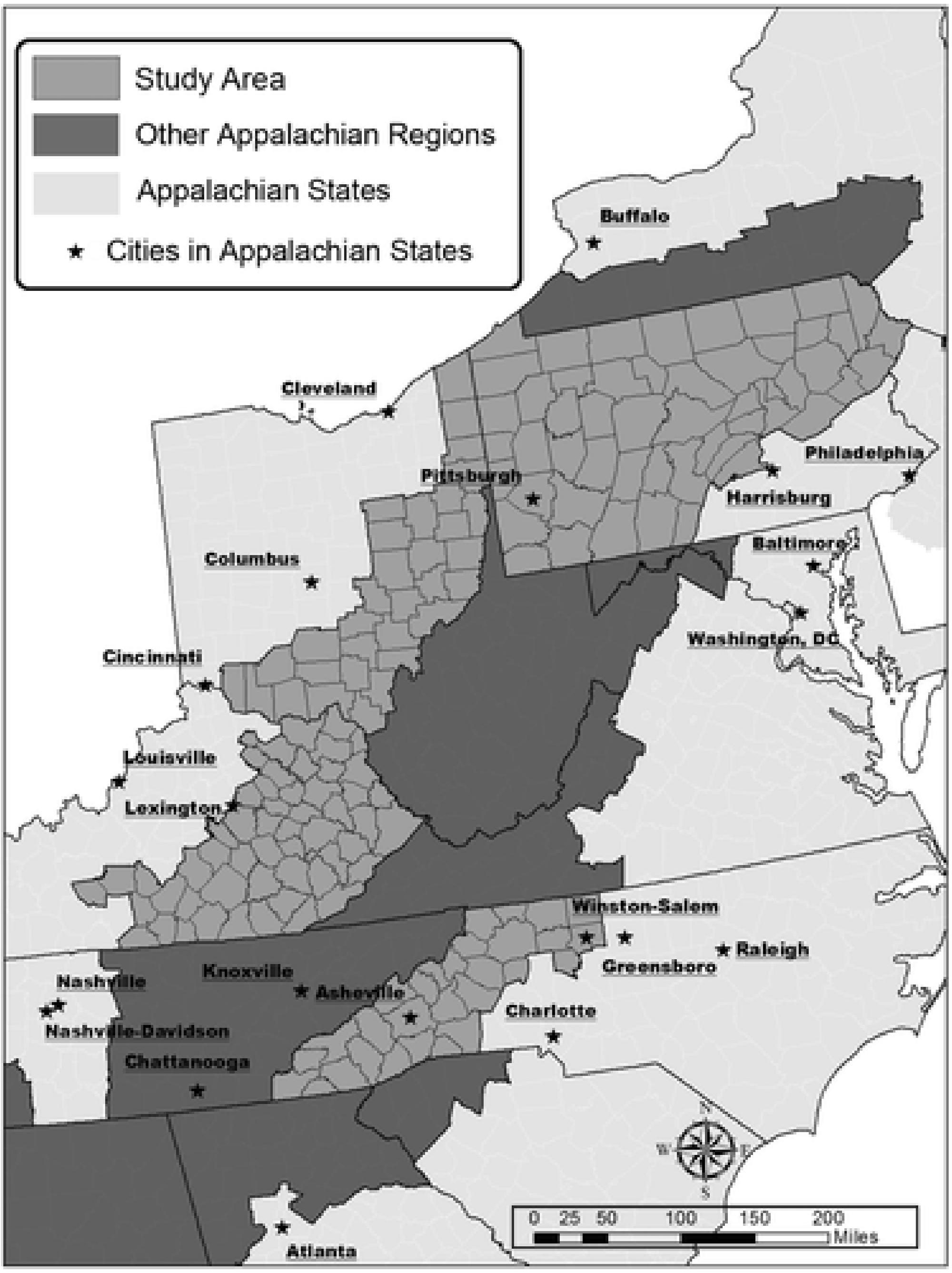

References

1. Janes H, Dominici F, Zeger S. On quantifying the magnitude of confounding. Biostatistics **11**(3), 572–582 (2010)
